# Restoration and Management of Healthy Wetland Ecosystems

**DOI:** 10.1155/2017/1874604

**Published:** 2017-04-19

**Authors:** Dong Xie, Qiang Wang, Zhongqiang Li, Roger Paulo Mormul, Liangdong Zhu

**Affiliations:** ^1^Co-Innovation Center for Sustainable Forestry in Southern China, Nanjing Forestry University, Nanjing 210037, China; ^2^School of Ecological and Environmental Science, East China Normal University, Shanghai 200062, China; ^3^School of Resources and Environment, Hubei University, Wuhan 430062, China; ^4^Biology Department, State University of Maringá, 87020-900 Maringá, PR, Brazil; ^5^Vaasa Energy Institute, Vaasa University, 65101 Vaasa, Finland

Wetland ecosystem is one of the most important ecosystems in the world. However, with the large-scale urbanization and rapid economic development, the wetland ecosystem is facing increasing ecological and environmental issues including eutrophication, water pollution, biodiversity loss, and ecological function decreases. Therefore, improving and restoring wetlands and creating healthy wetland ecosystem are the pressing issues these days.

Healthy ecosystem means not only ecological health itself, but also the long-term maintenance of healthy human population and the sustainable promotion of social-economic development. Healthy wetland ecosystem would provide not only suitable habitats for wildlife but also important ecosystem functions for local sustainable development. To create healthy wetland, two aspects are mainly included: one is restoration and remediation of degraded wetland ecosystems and the other is protection and management of wetland ecosystems which have not yet been disturbed, so that these wetlands can undergo benign development.

The restoration and remediation of the degraded wetlands are the most important issues of wetland researches and have developed rapidly with the development of wetland restoration theories in recent years. Our understanding of how wetland ecosystem changes is becoming more deeper than any time in human history, but descriptions of the variety and severity of changes have been repeatedly stated for decades now, yet the alarming trends continue. Similarly, wetland management traditionally derives its knowledge base from the fields such as aquatic chemistry and biology and hydrology. However, this disciplinary training does not equip wetland managers for the challenge of addressing the drivers of ecosystem change as described above, the societal processes that produce the need for more food and more water and land use change.

In this special issue, we focused on restoration and management of healthy wetlands. There are 3 aspects and 11 papers on biodiversity protection, hydrology management, and habitat restoration in the current special issue. For biodiversity protection, W. Du et al. monitored the composition and biomass of aquatic vegetation in the Poyang lake; H. L. Clipp et al. surveyed winter water bird community composition in West Virginia, USA; C. Yin et al. monitored the* Microcystis* biomass changes at Meiliang Bay, Lake Taihu, China; Y. Yuan et al. researched the effect of mowing on the competition of* Phragmites australis* and* Spartina alterniflora* in the Yangtze Estuary; Y. Zhang et al. monitored migratory shorebird responses to prey distribution in a large temperate arid wetland, China; Z. Ge et al. studied that emerged community enhanced the enrichment of nitrogen and phosphorus in the wetland. For hydrology management, Y. Zhang et al. studied the long-term relationship between precipitation and aquatic vegetation succession in east Taihu Lake, China. X. Chen et al. simulated the effect of artificial water transfer on carbon stock of* Phragmites australis* in the Baiyangdian wetland, China; N. Yang et al. studied the effect of hydrologic alteration on the community succession of macrophytes at Hanjiang River, China. For habitat restoration, J. Cao et al. studied improvement of urban water environment in Eastern China; Y. Li et al. studied a carbon cycle model for the social-ecological process in coastal wetland, East China.

The restoration and management of healthy wetland ecosystem mainly adopt biological, ecological, and engineering technology, gradually restore the structure and function of degraded wetland ecosystem, and finally reach the self-sustaining state of wetland ecosystem. The core messages and directions of this special issue would ameliorate the ecological and environmental effects on wetlands; at least, they might reflect some questions or problems, which grabbed the researches and managers attention.

*Dong Xie* *Qiang Wang* *Zhongqiang Li* *Roger Paulo Mormul* *Liangdong Zhu*

